# Male reproductive success is not strongly affected by phenological changes in mate availability in monoecious *Sagittaria latifolia*

**DOI:** 10.1098/rsos.231117

**Published:** 2023-09-27

**Authors:** Allison Kwok, Samantha Stephens, Marcel Dorken

**Affiliations:** ^1^ Environmental and Life Sciences Graduate Program, Trent University, Peterborough, Ontario, Canada K9L 0G2; ^2^ Department of Biology, Trent University, Peterborough, Ontario, Canada K9J 7B8

**Keywords:** dichogamy, flowering phenology, mating environment hypothesis, monoecy, sex allocation, protogyny

## Abstract

Many plants express their female and male sex roles at different times (dichogamy), with important consequences for mating. Dichogamy can yield mate limitation via biased floral sex ratios, particularly at the beginning and end of the flowering season when many plants simultaneously function as the same sex. This form of mate limitation should be reduced if plants adjust their allocations to female versus male sex functions in a manner that tracks seasonal variability in mating opportunities. For example, under protogyny (i.e. dichogamy with female function expressed first) plants with male-biased sex expression should have enhanced mating opportunities early in the flowering season as other plants begin to flower (in female sex phase). We quantified seasonal changes in sex allocation, patterns of mate availability and realized siring success in a population of protogynous *Sagittaria latifolia*. Our results were consistent with previous findings that seasonal changes in sex allocation should compensate for lost mating opportunities under the temporally variable mating environments caused by dichogamy. However, patterns of siring success in the population were inconsistent with this interpretation. We suggest that realized siring success might depend more strongly on spatial than on temporal aspects of mate availability.

## 1. Introduction

The timing of life-history events has important consequences for organismal fitness via effects on reproduction, survival and growth. For plants, flowering time affects fitness via effects on mate availability [1]. However, there are important constraints on the timing of flowering [2,3], and in particular the timing that floral organs associated with female function (i.e. the gynoecium) versus those associated with male function (the androecium) are actively engaged in reproduction. On one hand, and all else being equal, mass flowering should maximize mate availability. On the other hand, because most plants are hermaphroditic, mass flowering can have negative consequences for mate acquisition from the repeated transfer of pollen grains among flowers of the same plant (i.e. geitonogamy), deleteriously affecting reproductive fitness via the female and male functions of plants [4]. For example, in mass flowering *Rhododendron ferrugineum* outcrossing was determined by the local availability of mates, with high selfing rates when pollinators transferred self pollen within patches of low mate availability [5].

Many plants can avoid the negative consequences of self pollen transfer between flowers via the temporal segregation of their female and male function (i.e. dichogamy). Dichogamy separates the timing of pollen export and pollen receipt, thereby reducing self-interference within flowers (e.g. reduced pollen export and/or increased self-fertilization via the presentation of pollen and receptive stigmas in the same flower at the same time; [6]) and reducing geitonogamous pollen transfer (e.g. by reducing the frequency that pollinators move between flowers in complementary sex phases [7]). This reduction in geitonogamous pollen transfer is expected whether dichogamy is synchronous (no temporal overlap in the expression of female and male sex functions within plants) or asynchronous (some temporal overlap in the expression of sex functions) [7]. The reduction in self-interference from dichogamy can improve the efficiency of pollen export with fitness benefits to plants via their male function (i.e. as pollen donors; [8]). There are two main classes of dichogamy: protandry (male function is expressed before female function) and protogyny (female function is expressed before male function). Dichogamy is a widespread feature of outcrossing angiosperms and appears to occur in most species [9].

**Figure 1.**
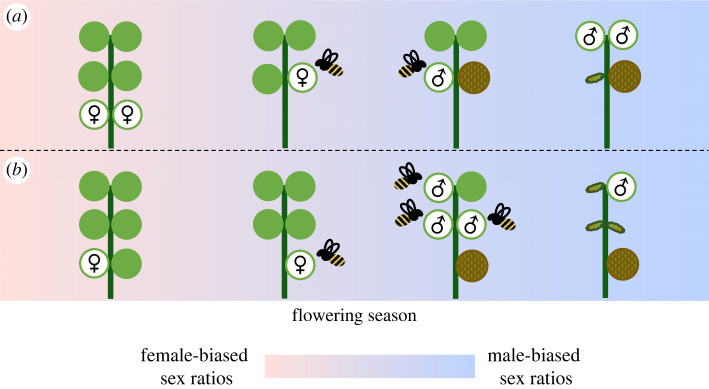
Under protogyny, floral sex ratios are female-biased at the beginning of the season and male-biased at the end of the season, with potential consequences for mate availability. Each panel is composed of maturing inflorescences with flower buds (green circles), gradually maturing female or male flowers (open circles with female or male symbols), and either maturing fruits (spotted green and brown circles) or senesced male flowers. (*a*) A plant that allocates resources equally between its female and male sex functions may lose mating opportunities due to flowering synchrony with other plants in the population. (*b*) By adjusting sex allocations to its male function earlier, a plant might have additional mating opportunities to transfer pollen to open female flowers than the plant in (*a*). Mate limitation is indicated by flowers that are not visited by pollinators.

The positive effects of dichogamy on mating are counterbalanced by skewed floral sex ratios that limit mate availability [10]. Under protogyny, the first plants to flower may be pollen limited if most other plants are also expressing the female sex phase. Similarly, the last plants to flower are also expected to be mate limited—in this case by the frequency of plants expressing male sex phase. However, these negative effects of mate limitation under dichogamy should be mitigated if plants adjust their sex allocations across the flowering period such that (gametophytic) sex ratios are re-balanced [1]. For example, plants in protogynous populations with increased allocations to male function early in the flowering period (and decreased allocations to male function towards the end of the flowering period) have greater mating opportunities than plants that do not adjust their sex allocations (figure 1). These enhanced mating opportunities arise because biased floral sex ratios under dichogamy favour plants that express the minority sex. Accordingly, adjustments of sex allocation towards the minority sex at any time in the season are expected to enhance mating success via a plant's female and/or male sex functions [1].

That plants might adapt to temporal changes in floral sex ratios via evolutionary adjustments to floral sex allocations in early- versus late-developing flowers is referred to as the mating environment hypothesis [11]. Empirical support for the mating environment hypothesis exists in the form of evidence that the female function of plants (e.g. fruit set) in protandrous populations [12], but whether benefits also accrue to male function via enhanced siring has not been demonstrated empirically. There is therefore a gap in the testing of the mating environment hypothesis, which is built around the idea that adjustments to floral sex allocations over time in dichogamous plants yield enhanced pollen transfer probabilities [1].

Here, we test the hypothesis that variation in patterns of sex allocation over the duration of the flowering season affect reproductive success via male function (i.e. siring success) in protogynous *Sagittaria latifolia*. Flowers of *S. latifolia* are unisexual and in monoecious populations inflorescences produce a combination of female and male flowers [13]. Flowers open for a single day and inflorescences are synchronously protogynous—female flowers occupying basal positions of the inflorescence open first and after female phase is over male flowers begin to open. As a result, on a given day, inflorescences are functionally unisexual. Because of monoecy, adjustments to sex allocation occur at the inflorescence level—a given number of male flowers, plants can increase or decrease their sex allocation via adjustments to the production of female flowers (or vice versa [14,15]). If protogyny affects the availability of female flowers for outcross-pollen transfer, we expected (1) that opportunities for siring will change over the flowering season. In particular, these opportunities should decline towards the end of the season as fewer new inflorescences begin to flower (and therefore no new female flowers become available). We further expected (2) that plants adjust their sex allocations to reflect these changes in the availability of female flowers, with greater allocations to male flower production early in the season when there are many open female flowers. Finally, we expected (3) that the joint effects of changes in siring opportunities over the season and changes to floral sex allocations affected patterns of siring. In particular, we expected that pollen-transfer probabilities—estimated from temporal changes in the availability of mating partners and competition for mating events [1]—were positively associated with siring success. To evaluate these expectations, we tracked flowering phenology and inflorescence-level sex allocations over an entire flowering season to calculate pollen-transfer probabilities and conducted parentage analysis to estimate siring success via male function.

## 2. Methods

### 2.1. Study species

*Sagittaria latifolia* Willd. is an emergent clonal aquatic plant, commonly found in wetland habitats across North America. Daughter ramets are produced at the ends of stolons during the growing season and can be vegetative or reproductive [15]. Corms are produced at the end of stolons and are the perennating structures [16]. Across the species range, populations of *S. latifolia* can be either dioecious or monoecious [17]. In monoecious populations of *S. latifolia*, flowers are produced on racemes with three flowers at each node. Female flowers occupy basal nodes and male flowers occupy distal nodes. Flowers open basipetally, both female and male flowers remain open for a single day, and nodes with male flowers mature later than nodes with female flowers, resulting in inflorescences that are synchronously protogynous [15]. Female flowers tend to open simultaneously, while the opening of male flowers is staggered [18]. Male flowers tend to be larger, while female floral displays are larger [19]. Flowering in *S. latifolia* in southern Ontario occurs from late June through to mid-September and flowers are pollinated by a variety of pollinators, including solitary and social bees, wasps, flies, beetles and lepidopterans, that forage for both pollen and nectar [19]. Larger flower size and floral display size have been shown to positively affect pollinator visitation [19]. Plants are fully self-compatible [13,20], but temporal separation of female and male flowers makes self-fertilization within a ramet unlikely [13,21]. However, because of clonality, selfing between flowering ramets of the same genet can occur, and selfing rates in monoecious populations can vary substantially (range in selfing rates across six monoecious populations: 0.13–0.63 [13]).

### 2.2. Study site and phenological sampling

To examine flowering phenology and sex allocation in *S. latifolia*, a monoecious population in Peterborough, ON (44° 20′ N, 78° 18′ W) was visited daily for the duration of the flowering season (between 18 July 2013 and 28 August 2013). The study site was in a shallow part of Thompson Creek in Meadowvale Park, Peterborough Ontario, covering approximately 25 × 60 m, and separated from other populations of *S. latifolia* by at least 100 m. Flowering ramets were distributed throughout the site, with some ramets grouped in patches and others more spatially isolated. The phenological data were collected from the same population as in Stephens *et al.* [21] and Holt *et al*. [22]. Each day, newly-opened inflorescences were tagged, and flowering dates were recorded for a total of 943 flowering ramets. The numbers of female and male flowers open on each ramet were also recorded. A sample of leaf tissue from the youngest leaf of each flowering ramet was collected in labelled coin envelopes and dried using Sorbead silica beads (eCompressedair, OK, USA). Once dry, the leaf tissue was stored at −20°C . Developing fruits in the population were monitored and collected as they matured. A total of 757 fruits were collected from 220 ramets (average = 2.7 fruits collected per ramet). Our goal was to sample one seed per fruit for paternity analysis, however, not all fruits were fully mature at the time of collection, and we were therefore unable to extract viable DNA from all samples; in the end 358 seeds from 154 ramets were used for genetic analyses.

### 2.3. DNA extraction and SSR genotyping

We assigned ramets to genets and sires to seeds so that variation in realized siring success could be assessed at the whole-plant (genet) level. These procedures are outlined in detail in Stephens *et al*. [21]. Briefly, genomic DNA extraction for genet assignment involved approximately 5 mg of dried leaf tissue, homogenized using a MM 300 Retsch mixer mill (Haan, Germany). DNA was extracted using the E.Z.N.A. Plant Extraction kit (Omega Bio-Tek Inc, GA, USA) and eluted to a final volume of 100 µl. To extract seed DNA, seeds were soaked in distilled water for 24 h, after which they were separated from the surrounding fruit tissues and the seed coat. Seed DNA was extracted using the QuickExtract Plant Seed DNA Extraction Solution (Lucigen, Madison WI) following the manufacturer's protocol, and eluted to a final volume of 50 µl.

Samples were genotyped at seven microsatellite loci (SL06, SL09, SL21, SL30, SL74, SL75 and SL88; [23]). Fluorescent labels FAM (SL06, SL27, SL30, SL74 and SL75) and HEX (SL21, SL09 and SL88) were applied to the forward primers. Polymerase Chain Reaction (PCR) amplifications were performed as either singleplexes (SL06, SL27 and SL75) or as multiplexes (SL09/SL74/SL88; SL21/SL30). The singleplexes were performed in a total reaction volume of 10 µl including approximately 10 ng of DNA, 1 × PCR buffer, 2 mM MgCL2, 0.15 mM of dNTPs, 0.3 mg ml^−1^ BSA, 0.2 µM of each forward and reverse primer, 0.5 U/reaction of Taq DNA polymerase (Invitrogen, Waltham, MA, USA) and additional ddH2O. The singleplex PCR conditions consisted of initial denaturation step of 94°C for 3 min; 30 cycles of 94°C for 30 s, 60°C for 30 s, and 72°C for 45 s; and a final extension of 72°C for 45 min. Multiplex reactions were conducted in total reaction volumes of 10 µl. Each 10 ul SL21/30 reaction included approximately 10 mg of DNA, 1 × PCR buffer, 1.3 mM of MgCl2, 0.3 mM of DNTPs, 1 mg ml^−1^ BSA, 0.1 µM each of SL21 forward and reverse primers, 0.19 µM each of SL30 forward and reverse primers, 0.5 U GoTaqFlexi DNA polymerase (Promega, Madison, WI, USA) and additional ddH2O. For the SL09/74/88 multiplex, the reaction volume of 10 µl included 1 × PCR buffer, 1.6 mM MgCl2, 0.3 mM DNTPs, 1 mg ml^−1^ BSA, 0.2 µM each of SL88 forward and reverse primers, 0.1 µM each of SL09 forward and reverse primers, 0.16 µM each of SL74 forward and reverse primers, and 0.5 U GoTaqFlexi DNA polymerase (Promega, Madison, WI, USA) and additional ddH2O. The PCR conditions for the multiplex reactions were as follows: initial denaturation at 94°C for 2 min; 35 cycles of 94°C for 45 sec, annealing temperature of 57°C for Sl21/30; 56.5°C for Sl09/74/88 for 45 sec, 68°C for 45 sec; and a final extension of 68°C for 8 min. Each PCR included a negative control using ddH2O and 10% of samples were re-amplified to ensure consistency. Amplifications were carried out on a MasterCyclerepGradient thermocycler (Eppendorf, Hamburg, Germany). PCR products were screened using gel electrophoresis in a 1.5% agarose gel and 10% SYBRGreen (Sigma Aldrich, St. Louis, MO, USA) at 90 V; fluorescently labelled products were scored using an automated sequencer (Applied Biosystems 3730 DNA analyzer) with a ROX size standard (Applied Biosystems, Waltham, MA, USA) and analysed using GeneMarker (SoftGenetics, State College, PA, USA).

### 2.4. Genet and paternity assignment

Using the SSR data we grouped ramets into multi-locus genotypes and then assigned them to clones (genets) using the RClone package (v. 1.0.1.1, [24]) in R (v. 3.4.4; [25]) following procedures outlined in Holt *et al*. [22] and Stephens *et al*. [21]. Ramets with multi-locus genotypes (MLGs) that differed by up to one allelic difference at any locus were grouped into multi-locus lineages (MLLs) using the MLG_list and MLL_generator2 functions from the RClone package (for additional details on MLL assignment, see Holt *et al*. [22]). This procedure yielded 210 MLGs, and 169 unique MLLs (for more details, see [21]).

Genets (MLL) comprised between 1 and 35 ramets. The majority of genets (40%) were made up of single ramets, with a few large genets. Assignment of paternity to one or more of these genets was conducted using a combination of Cervus (v. 3.0.7; [26]) and COLONY2 (v. 2.0.6.5; [27–29]). Paternity analysis was conducted using MLG assignments and these MLG-level assignments were then mapped to the corresponding MLL. To conduct the analysis, we used the full-likelihood method for determining the most likely full- and half-sib families using COLONY2. Runs in COLONY2 were made with medium-length runs and with high-likelihood precision, incorporating per locus stochastic error rates that were determined by comparing expected and observed heterozygosity in parental genotypes per locus using Cervus. Ploidy was set as diploid and the mating system was set as polygamous for both males and females, without inbreeding (parental inbreeding coefficients in monoecious populations are low, ranging between 0 - 0.2 from a sample of six populations; [13]). Because we knew the identity of the maternal parents for each seed, we specified maternal sibships for the analysis. Of the 358 seeds that were genotyped 312 were assigned paternity to one or more MLGs. The seeds had siring probabilities ranging from 0.01 to 0.94 (mean = 0.25 ± 0.22 s.d.), the majority of seeds (72%) were assigned to sires with 95% or greater confidence, with 24 seeds having siring probabilities with less than 10%. A total of 52 MLLs were assigned as sires to one or more seeds.

### 2.5. Male reproductive success

The MLG assigned as the most probable paternal parent of a seed was treated as the sire and for any given seed the probability of paternity *π* was used to estimate total siring per MLL. These probabilities were summed across all the seeds assigned to that MLL as the paternal parent such that total reproductive success via male function *RS^m^* per MLL *i* was estimated as:

$$RS_{i}^{m}=\underset{j}{\sum }\pi_{ijk},$$

where *π* is the probability that each genotyped seed *j* was sired by MLL *i* on pollination date *k*. Self-fertilized seeds were excluded from calculations of $RS_{i}^{m}$.

### 2.6. Mating opportunities

The calculation of pollen transfer probabilities [1] yields a numerical description of opportunities for plants to obtain mates via their male function (see also [30]). Our approach involves a minor modification of the one taken by Brunet and Charlesworth [1]. In their study the focus was on the probability of pollen transfer for flowers occupying different positions in a sequence (and see Brookes & Jesson [12]). Here, the focus is on the probability of pollen transfer for ramets with male flowers that are open on a given day in the flowering period. We refer to the metric that describes opportunities for pollen transfer as the index of pollen transfer opportunity (the metric is not bound by 0 and 1). We estimated total relative pollen transfer opportunities per genet from matrices of female and male flower production per day for the ramets comprising a genet. We first calculated the daily fraction of open male flowers per ramet as:

$$f_{j}^{m}=\frac{N_{j}^{m}}{\sum_{l\neq j}^{n}N_{l}^{m}},$$

where $N_{j}^{m}$ corresponds with the total number of open male flowers on a ramet *j* and $N_{l}^{m}$ corresponds with the number of open male flowers on the *n* other ramets on that day. To estimate the relative pollen transfer opportunity per ramet, we multiplied the fraction *f^m^* by the total number of female flowers ($N_{i}^{f}$) open on the same day on all non-self ramets *i* (i.e. on all ramets other than those that were part of genet *g*):

$$K_{ij}=f_{\;j\subseteq g}^{m}\times \overset{n}{\underset{i\notin g}{\sum }}N_{i}^{f}.$$

Daily values of *K_ij_* were summed across all the *j* flowering ramets from the same genet *g* to estimate the per genet pollen transfer opportunity as: $K_{g}=\sum_{\;j\in g}K_{ij}$.

### 2.7. Seasonality of sex allocation

Because *S. latifolia* is monoecious and inflorescences include a combination of female and male flowers, the smallest unit of organization at which sex allocation is expressed is the inflorescence. To examine whether patterns of allocation to male and female flowers varied over the flowering season, we calculated the proportion of male flowers per inflorescence (referred to here as the inflorescence sex ratio) (*n* = 298 inflorescences) and used this as the measure of sex allocation. We used a generalized additive mixed model (GAMM), as implemented by the gamm4 function from the gamm4 package (v 0.2-6 [31]) in R (v. 4.3.1; [25]). GAMM was chosen because we had no *a priori* expectation regarding linear versus nonlinear changes in sex allocation over time. The relaxed assumptions of linearity in GAMMs involve the calculation of nonlinear smoothing splines, enabling the estimation of linear or nonlinear associations between response and predictor variables [32]. A significant parameter estimate for the smoothing function indicates that a nonlinear model provides a better fit. In the GAMM, inflorescence sex ratio (a composite variable of the number of female and male flowers) was the response variable and the date of first flowering per inflorescence was the predictor variable. The model was specified using a binomial error distribution and a logit link function. Because inflorescences and MLLs were sampled across multiple days, inflorescence and MLL were specified as random effects, with inflorescence nested within MLL. The default base function of thin plate regression spline was used to estimate the degree of smoothing because of the small number of predictor variables.

### 2.8. Seasonal changes in mating opportunities

To evaluate seasonal changes in mating opportunities via the male function, we again used a GAMM. For this model, daily pollen transfer opportunity was the response variable and date was the independent variable and treated as a fixed effect. Because pollen transfer opportunities were calculated at the genet level (*n* = 123 genets) and repeated sampling of MLLs occurred over multiple dates, MLL was included as a random effect. The GAMM was specified using a Gaussian error distribution with an identity link function and calculated using the gamm4 function from the gamm4 package in R. The gam.check function from the mgcv package [33] was used to ensure that this model and the one described in the previous paragraph (changes in infloresence-level sex ratios over time) were not over-fitted. For both GAMM models we examined whether the inclusion of a smoothing function improved model fit by comparing GAMM results with those from linear and generalized linear mixed-effects models with the same dependent and independent variables using the lmer functions from the lmerTest pacakage (v 3.1-3 [34]) and glmer functions from the lme4 package (v 1.1-33 [35]) in R and then compared AIC values between models [36]. In both cases, the smoothing term was associated with a significant parameter estimate and the GAMMs were assigned lower AIC values than the linear models. Accordingly, here we report the GAMM results and linear model results are reported in supplementary materials (electronic supplementary material, tables S1. Sex allocation GLME and S2. Pollen transfer opportunity LME).

### 2.9. Seasonal changes in mating opportunities and siring success

To evaluate the prediction that siring success was positively associated with pollen transfer opportunity, we used a linear mixed-effects (LME) model using the lmer function, with siring success (*n* = 39 genets) as the dependent variable and pollen transfer opportunity and siring date as independent variables, each treated as fixed effects. The siring success data were right skewed with many near-zero values. Accordingly, siring success was log_10_-transformed. As with previous analyses, MLL was treated as a random effect. We used step-wise model selection to determine the effect of siring date and pollen transfer opportunity as separate predictors and together [36].

## 3. Results

### 3.1. Flowering phenology

**Figure 2.**
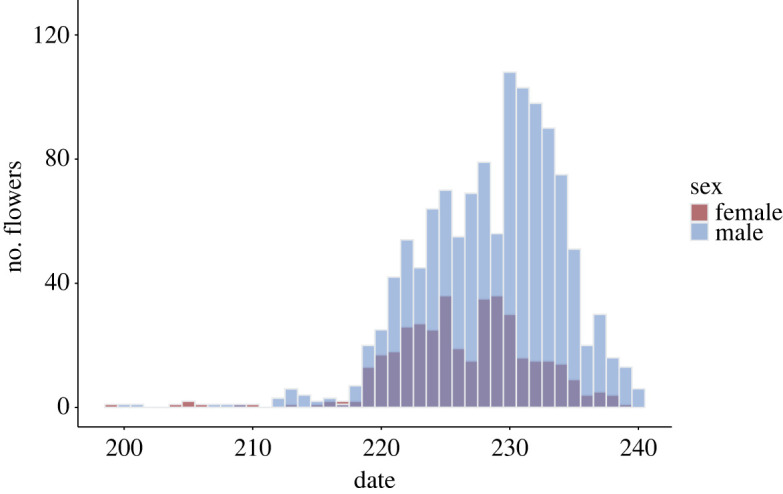
Total numbers of open female and male flowers per day in a monoecious population of *Sagittaria latifolia* from July 18th (day 199) to August 28th, 2013 (day 240).

Flowering started on 18 July 2013 (Julian date = 199) and ended on 28 August 2013 (day 240). Only eight inflorescences had open flowers over the first 12 days of the season. Starting August 3rd (day 215) a minimum of two different genets flowered each day with at least one genet in male phase and another in female phase until August 28th (day 239; i.e. mating events were continually possible over this period). Peak flowering, the day with the greatest number of open inflorescences, occurred on August 18th (day 230; figure 2 and see electronic supplementary material, figure S1. Phenogram of sex allocation). Peak male phase (254 male flowers on 107 inflorescences from 67 genets) occurred on August 18th (day 230), one day after peak female phase on August 17th (day 229; 117 female flowers on 36 inflorescences from 25 genets).

### 3.2. Sex allocation

**Figure 3.**
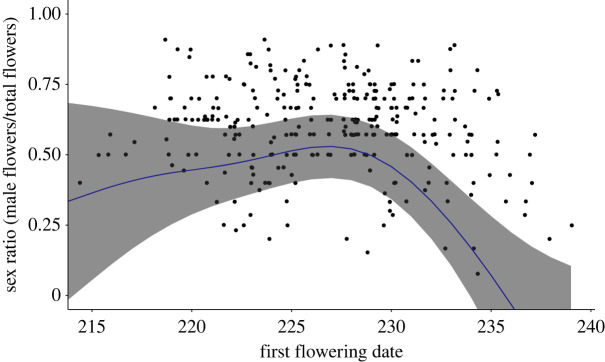
First flowering date and the corresponding inflorescence sex ratio (proportion of male flowers) for each ramet in a monoecious population of *Sagittaria latifolia* between August 3rd (day 215) to August 28th, 2013 (day 240). The solid line represents the estimated trend in the date of first flowering from a generalized additive mixed-effects model and the shaded area represents the 95% confidence intervals.

After August 3rd (day 215; i.e. during the period of continuous flowering), inflorescence-level sex allocations tended to be male-biased (figure 3; electronic supplementary material, figure S2. Inflorescence sex allocation for full flowering season). By contrast, after peak flowering (August 18th, day 230), inflorescences tended to be female-biased. This change in inflorescence-level sex allocations was associated with a significant nonlinear association between date and floral sex ratios (table 1 model A). The smoothing term of the nonlinear model was significant and the GAMM provided a better fit compared to the GLME model (electronic supplementary material, table S1. Sex allocation GLME).

**Table 1.** Generalized additive mixed-effects models (GAMMs) of temporal changes in inflorescence sex allocation (A) and pollen transfer opportunity (B) in a monoecious population of *Sagittaria latifolia*. Values are the parameter estimates, and associated *t*- and *p*-values for the modelled average sex allocation (model intercept), the effective degrees of freedom (*edf*) and associated test statistics (model A: *χ^2^*, model B: *F*-test) for the smoothing term, and model Akaike Information Criterion values (AIC). Note: Significance *p < 0.05, **p < 0.01, ***p < 0.001.

model	term	estimate	s.e.	*t*	*p*	*edf*	*χ*^2^/*F*	*p*	AIC
A	(intercept)	0.42	0.04	10.41	<0.001***				1058.25
	s(first flowering date)					3.3	13.19	<0.01**	
B	(intercept)	0.24	0.03	6.97	<0.001***				11423.11
	s(date)					7.69	72.73	<0.001***	

### 3.3. Pollen transfer opportunity

**Figure 4.**
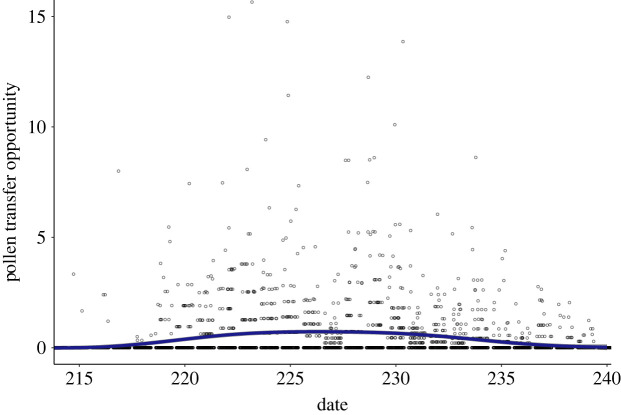
Temporal trend of mating opportunities per ramet in a monoecious population of *Sagittaria latifolia* between August 3rd (day 215) to August 28th, 2013 (day 240). Mating opportunities were measured as the daily pollen transfer opportunity (PTOs) per MLL. The solid line indicates the daily trend in PTOs from a generalized additive mixed effect model. PTO values (open dots) are jittered so that overlapping values can be more easily seen.

In the first ten days of the flowering season there were so few flowering plants that pollen transfer opportunities were at or close to zero (electronic supplementary material, figure S3. Pollen transfer opportunity). After July 28th (day 219) pollen transfer opportunity values began to increase, with male-phase plants having the greatest opportunities for siring between Aug. 7^th^–18th (day 220 to 230; figure 4). After August 18th, pollen transfer opportunities began to decrease, indicating declining siring opportunities towards the end of the season. These changes in pollen transfer opportunity over time were associated with a significant smoothing term for flowering date in the GAMM, indicating a nonlinear association between pollen transfer opportunity and date (table 1 model B; cf. electronic supplementary material, table S2. Pollen transfer opportunity LME).

### 3.4. Siring success

**Figure 5.**
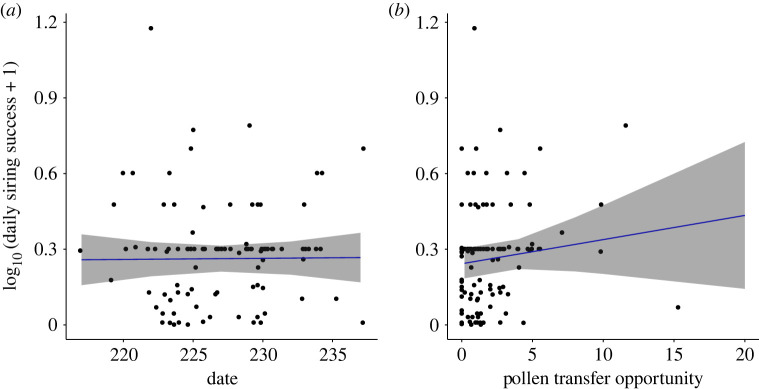
Linear regression of daily siring success (log_10_ transformed) with (*a*) siring date and (*b*) pollen transfer opportunity per genet (MLL) in a monoecious population of *Sagittaria latifolia*. Solid lines are the least-squares regression lines and the shaded areas represent the 95% confidence intervals.

Patterns of siring success did not vary over time (figure 5*a*) and the association between siring success and date was not significant (*F*_1,96.13_ = 1.03 × 10^−2^, *p* = 0.92, table 2 model A; figure 5*a*). There was also no relationship between siring success and pollen transfer opportunities (*F*_1,100.03_ = 1.41, *p* = 0.24; table 2 model B; figure 5*b*), contrary to our prediction that pollen transfer opportunities reflected realized opportunities for obtaining mates as a male parent.

**Table 2.** Linear mixed effects models of siring success in a monoecious population of *Sagittaria latifolia*. Independent variables were pollen transfer opportunities (PTO; A) and siring date (B). Values are the parameter estimates, and associated *t*-test and *p*-values. We also report measures of overall fit for each model, including *F*-tests and their numerator and denominator degrees of freedom, and model AIC values. Note: Significance *p < 0.05, **p < 0.01, ***p < 0.001.

model	term	estimate	s.e.	*t*	*p*	*F*	*df*	*p*	AIC
A	(intercept)	0.16	0.97	0.17	0.87	0.01	1, 96.13	0.92	−39.21
	date	4.36 × 10^−4^	4.29 × 10^−3^	0.10	0.92				
B	(intercept)	0.24	0.03	7.88	<0.001***	1.41	1, 100.03	0.24	−41.87
	PTO	9.58 × 10^−3^	8.10 × 10^−3^	1.19	0.24				

## 4. Discussion

Changes in inflorescence-level sex allocations over the course of the flowering season coincided with changes in mating opportunities via male function, but not realized siring success in the population of *S. latifolia* studied here. Early and mid-season inflorescences were male-biased, but inflorescences became increasingly female-biased towards the end of the season. The timing of these changes in sex allocation in the population occurred in the manner expected under the mating environment hypothesis: when plants began to flower at the start of the season (in female phase), plants tended to have male-biased allocations. Similarly, towards the end of the season as fewer new plants began to flower, plants became increasingly female-biased in their allocations. These changes in sex allocation should have affected mating opportunities through male versus female function: siring opportunities (measured as pollen transfer opportunity) were highest during peak flowering, coinciding with the period over which allocations were most strongly male biased. In so far as we have demonstrated that patterns of sex allocation change in a manner that should increase siring opportunities, our data are consistent with the mating environment hypothesis and provide a similar degree of support as previous tests of the hypothesis [1,11,12]. However, pollen transfer opportunities were not clearly associated with realized patterns of siring success, and so this study fails to provide direct support for the mating environment hypothesis. Processes not accounted for in this study might have had stronger effects on variation in siring success than seasonal changes in inflorescence-level sex allocations, including (1) sexual selection [37] and (2) spatially restricted pollen dispersal [21]. (3) Patterns of size-dependent sex allocation in *S. latifolia* also appear to be confounded with expectations from the mating environment hypothesis. Below we discuss how these alternative processes might have affected patterns of mating, obscuring the expected association between pollen transfer opportunities and siring success.

### 4.1. Sexual selection

The calculation of pollen transfer opportunities assumes that the transfer of pollen to female flowers is determined by a plant's production of male flowers. However, this assumption is violated if opportunities for pollen transfer are unequal. At least two processes can generate such unequal patterns of pollen movement. The first of these is sexual selection, defined as non-random variation in mating success [38] and has been shown to operate in the population studied here [36]. Previous studies have implicated variation in floral-display size as a potential mechanism underlying sexual selection in *S. latifolia* [39]. For other plants, the contributions of individual flowers to fitness can decline on a per-flower basis, even if total siring is greater for larger compared to smaller inflorescences [40]. However, smaller per-flower contributions to siring success on larger inflorescences may be caused by the transfer of self-pollen within inflorescences [39]—something that is not possible under synchronous protogyny in *S. latifolia*. Indeed, there appears to be a direct association between flower production and siring success [41]. Perhaps as a result of this linear association between male flower production and siring success, individuals with large male floral displays might have sired a disproportionate number of seeds in our study, regardless of seasonal changes in mate availability.

### 4.2. Spatially restricted pollen movement

The second process generating non-random mating is spatially restricted pollen dispersal. Realized patterns of pollen transfer are determined not only by the proportions of flowers in female versus male phase but also by the distances between mating partners. Pollination tends to occur between near neighbours [42–44] and to decline with distance [45]. Indeed, distances between mates combined with overlap in flowering time determine patterns of realized mating (e.g. *Echinacea angustifolia* [46]). However, the calculation of pollen transfer opportunities does not incorporate these spatial effects and might therefore miss an important aspect of mating in plants. Indeed, a previous study of *S. latifolia* found that realized patterns of pollen dispersal follows a negative exponential distribution, with mating subject to some degree of spatial restriction [21]. Studying the joint effects of phenology and spatial distributions may provide greater insight into realized mating opportunities in plants.

### 4.3. Size-dependent sex allocation

Sex allocation in plants can be strongly size-dependent and a variety of adaptive processes underlying patterns of size-dependent sex allocation have been proposed [47]. These mechanisms include changes in the expression of reproductive trade-offs between female and male function for plants of different sizes [48,49]; and changes in the strength of local mate competition and/or local resource competition [47,48] as plants increase in size. For monoecious populations of *S. latifolia*, larger ramets tend to produce more female flowers than smaller ramets [14]. However, the production of male flowers tends to be independent of ramet size, resulting in female-biased allocations for larger ramets [14]. This pattern of size-dependent sex allocation in *S. latifolia* has been interpreted as a mechanism to prevent the over-production of pollen in large shoots that might otherwise result in increased geitonogamous selfing [14]. Although we cannot rule out the operation of these processes in the population studied here, patterns of size-dependent sex allocation in *S. latifolia* are also consistent with expectations under the mating environment hypothesis. In particular, synchronous protogyny should favour male-biased allocations early in the flowering season (when ramets are small) and female-biased allocations later in the season (as ramets increase in size). Indeed, because ramets increase in size over the flowering season, size may be a reliable cue for adaptive adjustments in sex allocation under dichogamy.

## 5. Conclusion

Dichogamy—and synchronous dichogamy in particular—has the effect of limiting the availability of mating partners. Synchronized protogyny results in functionally unisexual inflorescences and, all else being equal, only half of the other shoots in the population are potential mating partners at any moment in time. These negative consequences of dichogamy are well known and might be more than compensated by the avoidance of self-fertilization and increased pollen-transfer efficiency via reduced sexual interference within flowers [10,50]. They are also thought to be compensated by seasonal changes to floral—or as for *S. latifolia*—inflorescence sex allocations that re-balance mating opportunities [1]. Previous studies have demonstrated that these seasonal adjustments to sex allocation can promote mating opportunities for dichogamous plants. Although our observational data largely mirror the results of those previous studies, paternity analysis failed to provide direct support for the mating environment hypothesis.

## Data Availability

All data and R code used in the analyses presented here are available on figshare at (http://dx.doi.org/10.6084/m9.figshare.23615817) [51]. Supplementary tables and figures are provided in electronic supplementary material [52].
